# Managing Mental Health in Pandemic COVID-19 and Movement Control Order

**DOI:** 10.21315/mjms2020.27.4.14

**Published:** 2020-08-19

**Authors:** Jamilah Hanum Abdul Khaiyom

**Affiliations:** Department of Psychology, Kulliyyah of Islamic Revealed Knowledge & Human Sciences, International Islamic University Malaysia, Kuala Lumpur, Malaysia

**Keywords:** pandemics, mental health, cognitive-behavioural therapy, mindfulness, spirituality

## Abstract

COVID-19 and the Movement Control Order (MCO) may trigger a ‘next wave’ of mental health problems. However, the relationship is not linear. Human psychology also has an impact on the outbreak. Thus, proper strategies to manage human psychology, especially mental health, is very important to break the vicious cycle. This article aims to discuss ways to manage mental health using cognitive-behavioural approaches, mindfulness and spirituality. Specific cognitive-behavioural and mindfulness strategies are listed and suggestions to return to the foundation of human existence are discussed. By practising the cognitive-behavioural, mindfulness, and spirituality strategies described, we may enhance our acceptance, optimism and commitment to prepare for a ‘new or renewed normal’.

## Introduction

Coronavirus disease 2019 (COVID-19) — an originally pneumonia of unidentified cause was first detected in Wuhan, China. About a month from it was first reported, the World Health Organization (WHO) on 30th January 2020 has declared the outbreak as a public health emergency of international concern (i.e. pandemic). After more than three months, the outbreak has affected more than 200 countries/areas/territories globally. As of 30th April 2020, the number of confirmed cases has risen to more than 3 million and more than 200,000 people have died (1).

Due to these, many countries have exercised lockdown but the rules are not the same for each country. The Malaysian government has taken initiative by asking the citizens to stay indoors and the Movement Control Order (MCO) was exercised beginning from 18th–31st March 2020, and was further extended until 12th May 2020. The order was based on the Prevention and Control of Infectious Diseases Act 1988 and the Police Act 1967 (2).

The MCO consisted of six instructions, which included: i) Comprehensive prohibition of mass movements and gatherings including religious, sports, social and cultural activities; ii) Comprehensive restrictions on all Malaysians travelling abroad; iii) Restrictions on the entry of all foreign tourists and visitors into the country; iv) Closing of all public and private childcare, kindergarten, primary and secondary schools, and pre-university institutions; v) Closing of all public and private institutions of higher learning and skills training institutes nationwide; and vi) Closing of all government and private premises except those involved in essential services (2).

As of 28th April 2020, seven directives for an Enhanced Movement Control Order (EMCO) (i.e. a more rigorous MCO) were executed in high risk areas to curb the spread of COVID-19 and enable the authorities to trace cases actively, from house to house (3). MCO and EMCO may be further extended if the conditions worsen or are continuously unsafe for the public.

## COVID-19, MCO and Mental Health

Fear and anxiety are natural responses to COVID-19 (4, 5, 6). The contributing factors to these are a sense of uncontrollability and unpredictability (4). Research initiatives on the novel virus are still ongoing (e.g. the development of COVID-19 vaccine) and hence many of us may feel uncertain whether we can effectively save ourselves and our loved ones from the outbreak. According to the Reference Group for Mental Health and Psychosocial Support in Emergency Setting of Inter-Agency Standing Committee (IASC), emergencies are always stressful (7). Learning from the previous international public health emergency scenarios (e.g. smallpox, severe acute respiratory syndrome [SARS] and H1N1), some psychological effects include emotional distress, behavioural and cognitive impact and diagnosed psychiatric illnesses. The triggers of the psychological effects include trauma exposure, restricted movements, limited resources and information, and perceived personal and family risk (8). However, specific stressors in relation to COVID-19 are the risks of being infected and infecting others (7). Furthermore, fever or other common symptoms of health issues may be mistakened for COVID-19 and may lead to the worry of being infected (7). When appropriate care and support system are not in place, caregivers may increasingly feel worried for their children. This is also relevant for vulnerable individuals (e.g. elderly, people with disabilities). Their physical and mental health may be deteriorated when their caregivers are quarantined or isolated due to the COVID-19 (7).

During this period of MCO, the majority of us were asked to socially distance ourselves (i.e. prevent us from interacting closely and frequently). Meanwhile, some of us are being quarantined (i.e. an act of separation and restriction of movement of an individual who has been exposed to the disease as a measure to see if they become sick), and/or being isolated (i.e. an act to separate people who are sick from those who are not) (9). Social distancing, quarantine and isolation may trigger different types of emotions such as worry, fear, anxiety, frustration, boredom, loneliness and anger. For example, we may feel uncertain and frustrated about how long we will need to remain in this situation. We may constantly question our future and the possible reduced/loss of income, feel bored because of the inability to do what we normally do, feel lonely because we cannot meet our loved ones, and feel angry due to the loss of freedom experienced. Stigma or phobia to certain people/races/nationality (i.e. xenophobia) may also increase because we think that we may be exposed to the disease and being asked to observe MCO due to others’ negligence.

Apart from the above responses, the impact of COVID-19 and MCO may worsen when more intense psychological symptoms appear and the frequency becomes more consistent. Some of the symptoms are physiological in nature. Such conditions include headaches, body pain, stomach problems, poor sleeping patterns (i.e. too much or too little), and excessive crying. Other psychological symptoms may be talking less or avoiding others (i.e. even within the confines of social distancing); having difficulties concentrating; feeling confused, numb, helpless or depressed; feeling anxious or having panic attacks; feeling angry and having little patience; excessive eating, extreme intakes of alcohol, or taking prescribed drugs in excessiveness (10). However, most people usually cope well during natural disasters such as pandemics, but only minority of people will develop more intense psychological problems such as suicidality. The suicide rates may increase especially among the elderly due to social isolation, loneliness and the worry about being a burden on their caregivers (11).

## The Impact of Our Thinking and Behaviour on COVID-19 and MCO

Epictetus, the great Greek Stoic philosopher argued that external events are not worrying in nature, instead it is the way we perceive them that creates worry among us. In other words, it is not the COVID-19 or MCO are disturbing us, rather, the problem lies in our interpretation of their significance.

Steven Taylor, a Professor in Clinical Psychology and the author of ‘*the Psychology and Pandemics*’ argued that pandemics is essentially psychological phenomenon and are not merely about an infectious virus (12). In accordance with the wise statement by Epictetus, pandemics can be caused and contained by the way people think, feel and behave towards the current situation. To relate to the current situation, if people think that the virus (i.e. severe acute respiratory syndrome coronavirus 2 – SARS-CoV-2) is simply like other flu viruses and is not harmful, they may not feel cautious and decide not to cover their mouths while coughing, wash their hands regularly, and comply with the physical distancing and the observation of MCO regulations. Due to these psychological reasons, the virus will spread and become a pandemic.

As has been discussed previously, the relationship is not linear. COVID-19 may also impact human psychology. In order to break the vicious cycle, it is important to manage an individual’s mental health to curb the outbreak. This becomes more essential when epidemiologists have also agreed the ‘next wave’ of the COVID-19 pandemic is mental illness (13).

## Maintaining Mental Health Using Cognitive Behavioural Approaches, Mindfulness and Spirituality

Following the 2014 Ebola outbreak in the bordering countries of Liberia and Sierra Leone, South London and Maudsley National Health Services Foundation Trust (SLAM) were commissioned to conduct an effective and sustainable psychological intervention (Ebola Psychological Support Services [EPSS]) to address the psychological needs of the Sierra Leonean Ebola Treatment Centre (ETC) staff. The team developed a culturally appropriate group cognitive behavioural therapy (CBT) in a stepped-care approach and evaluated its effectiveness in treating anxiety, depression and functional impairment amongst a sample of former ETC staff. Based on the results, group CBT was found to be effective in improving the treatment outcomes and promising psychological intervention for health care workers who worked in the frontline during public health emergencies (14, 15, 16).

Today, in order to maintain and/or improve our mental health during the COVID-19 pandemic, World Confederation of Cognitive and Behavioural Therapies urged everyone to challenge unhelpful thoughts related to COVID-19 (e.g. negative predictions on the worst-case scenario) and take flexible, balanced perspectives, and long-term optimistic view about the situation. The world and humanities have survived many different types of world crises before and the COVID-19 pandemic too shall eventually pass (4).

Apart from the cognitive strategies, below are some of the behavioural strategies that are helpful to do:

- engage in helpful behaviours such as taking actions to protect ourselves by following the government’s advice (e.g. washing hands with soap regularly, covering mouth and nose when sneezing or coughing, observing physical distancing, and staying at home);
- engage in general self-care (e.g. manage sleeping time, exercise at home, eat a balanced diet with regular mealtime, avoid excessive caffeine or alcohol, avoid doing something that we may regret later);
- engage in enjoyable activities that make us feel pleasant every day (e.g. watching movies, cook a nice meal, and gardening);
- engage in problem-solving (e.g. how to reduce procrastination while working from home);
- engage in relaxation exercises (e.g. long diaphragmatic breathing that may calm our sympathetic nervous system and progressive muscular relaxations that may relief our physical stress and tension); and
- limiting the time to watch or listen to the news that may exacerbate our worry, only take in the amount of news we need to be informed (e.g. for some people, limiting news intake to one or two times a day can really make a difference).

Due to superior empirical evidence (17), many countries around the world are currently conducting effectiveness trials of cognitive behavioural therapy (CBT) for COVID-19. For example in the UK, the Homerton COVID psychological support (HCPS) pathway has been developed based on the EPSS to support frontline National Health Services (NHS) staff (18). Similar strategies could be seen in France where clinical trial on online CBT for stress disorders among health care workers involved in the care of patients has been registered (19). The cognitive behavioural interventions are not currently limited to health care workers but also for the general community. In Oman, for example, a 12-week randomised controlled trial has been registered to test the effectiveness of therapist guided e-therapy versus self-help therapy on psychological distress (20).

Cognitive behavioural therapy is not the only approach that is currently utilised to maintain or improve mental health. Mindfulness-based interventions are other promising approaches. Jon Kabat-Zinn, the founder of mindfulness-based stress reduction (MBSR), defines mindfulness as awareness that arises through paying attention, on purposes, in the present moment, non-judgementally. There are many forms of mindfulness activities and different mindfulness-based interventions (e.g. MBSR, mindfulness-based cognitive therapy, dialectical behaviour therapy, acceptance and commitment therapy, mindfulness self-compassion, and others) which may have used mindfulness activities differently. However, the current article would like to focus on using mindfulness and loving-kindness/compassion meditation during the pandemic. Tara Brach, the world-known meditation teacher in her famous book ‘*Radical Compassion*’ invites people to practise a short meditation called RAIN (21). There are four steps in RAIN as below:

- **R**ecognise: Recognise our feeling the moment and mentally whisper it (e.g. “Okay, I’m feeling anxious”).
- **A**llow: Allow the feeling to exist, without the need to fix, control or judge it. Just let it be there.
- **I**nvestigate: Inviting ourselves into the body and identify the source of anxiousness in the body. Try to find out how it feels and gently paying attention to it. Breath with it.
- **N**urture: Put our hand on our heart and offer a kind and soothing message to ourselves (e.g. “It’s okay to feel anxious, my heart. Thank you for trying to protect me.”)

To enhance the effectiveness of loving-kindness/compassion meditation, repeating certain phrases in our mind or softly whisper it, such as “may I be safe”, “may I be healthy” and “may my life unfold with ease” will be more helpful. The ultimate results of loving-kindness/compassion meditation do not only focus on individual selves but also for greater humanity and all living beings. As for how we wish good for ourselves, we are encouraged to widen the circle of caring, by wishing for the same thing for our loved ones, people we neutrally feel about, and for all living beings (including those who we may have a negative feeling with).

Does practising meditation only ends at good wishes? Certainly not! Substantial empirical evidence has found that small and continuous efforts in practising mindfulness and loving-kindness/compassion meditation may lead to: i) improved brain regions on emotional self-awareness, self-regulation, and attention (22); and ii) enhance care towards ourselves (23) and others (24, 25, 26). The keyword here is practise. By practising mindfulness and loving-kindness/compassion meditation during this pandemic, more altruistic behaviours may arise. This will make ourselves happier, by making other people happy.

Mindfulness and meditation are interconnected with spirituality. This is evident in many eastern traditions and Abrahamic religions. As COVID-19 may trigger a lot of boredom, suffering and grieving; returning to the foundation of human existence (i.e. spirituality) is worth to be considered. Spirituality is ‘that aspect of humanity that refers to the way individuals seek and express meaning and purpose and experience their connectedness to the moment, to self, to others, to nature, and to the significant or sacred’ (27). Substantial empirical evidence have found that spirituality is significantly associated with better mental health (28, 29). Looking at the current situation of COVID-19 and the lockdown that is taking place, returning to the significant, sacred, divine being or our Creator may offer us some relief. The question is “How?”

Different belief systems may have different ways and activities that can be done. However, general activities include exercising mindfulness and practising meditation. Apart from that, planning a solitary time by separating ourselves from other people may help us to search for the values and meaning of the current moment. In the time of MCO, finding a solitary time may be a struggle and will not be an easy option for many. However, having binary thinking of either “I will do it perfectly or I will never do it” may not help us to prepare ourselves for solitary time. The keywords are planning, arranging space, allocating time, communicate with people around us on the importance of solitary time for everyone, and doing it! It is a commitment for our self-care and act of self-compassion. When we do it, we model, and therefore allowing our significant others to imitate us. It is hoped that by returning to the foundation of our existence, we will be more accepting and grateful, more committed to making the best out of the current situation, more balanced and optimistic about the future, and be more prepared and able to cultivate a ‘new or renewed normal’.

## Conclusion

COVID-19 and MCO may impact our mental health. However, the relationship is not linear. Our psychology, especially our state of emotion, may trigger and contribute to the consequences of the outbreak. Managing our mental health is our utmost priority now to ensure the curve can be flattened, MCO can be lifted and COVID-19 can be defeated.
